# Advances and Limitations of Current Epigenetic Studies Investigating Mammalian Axonal Regeneration

**DOI:** 10.1007/s13311-018-0636-1

**Published:** 2018-06-08

**Authors:** Ilaria Palmisano, Simone Di Giovanni

**Affiliations:** Laboratory for Neuroregeneration, Centre for Restorative Neuroscience, Division of Brain Sciences, Department of Medicine, Imperial College London, London, UK

**Keywords:** Epigenetic, HDAC, HAT, Transcription, Axonal regeneration, Spinal cord injury, Nerve injury, DNA methylation, RNA

## Abstract

Axonal regeneration relies on the expression of regenerative associated genes within a coordinated transcriptional programme, which is finely tuned as a result of the activation of several regenerative signalling pathways. In mammals, this chain of events occurs in neurons following peripheral axonal injury, however it fails upon axonal injury in the central nervous system, such as in the spinal cord and the brain. Accumulating evidence has been suggesting that epigenetic control is a key factor to initiate and sustain the regenerative transcriptional response and that it might contribute to regenerative success versus failure. This review will discuss experimental evidence so far showing a role for epigenetic regulation in models of peripheral and central nervous system axonal injury. It will also propose future directions to fill key knowledge gaps and to test whether epigenetic control might indeed discriminate between regenerative success and failure.

**Electronic supplementary material:**

The online version of this article (10.1007/s13311-018-0636-1) contains supplementary material, which is available to authorized users.

## Introduction

Injury to the nervous system, such as following traumatic spinal cord and brain injuries, or a vascular insult such as stroke, causes loss of neuronal function leading to sensory, motor, autonomic or cognitive impairment. Successful compensatory axonal sprouting from intact neurons or regeneration from injured axons, i.e. regrowth of the injured axon to the original target is required for functional recovery. In mammals, axonal regeneration relies on the synergy between the presence of a permissive extracellular environment and the intrinsic growth capacity of the injured neurons [1–3]. While the mammalian peripheral nervous system (PNS) axons are able to regenerate to a certain extent, axons in the central nervous system (CNS) do not, due to the presence of glial inhibitory signalling, glial scar and to a limited neuronal intrinsic regenerative ability [4–6]. Interference with the inhibitory glial extra-neuronal cues has resulted into an improvement of axonal regrowth in the injured CNS [7–9]. However, this remains rather limited, and it has prompted the development of strategies aimed to enhance the poor neuronal intrinsic growth ability. While protein translation seems to play a role (reviewed elsewhere [10, 11]), the axon growth competence of neurons in both the PNS and CNS depends mainly on gene transcription, and on the capability to initiate an orchestrated transcriptional response to injury [12]. This ability declines during neuronal maturation and with aging [13–17]. The decline in regenerative ability is mainly due to changes in the transcriptional programme, which is affected by an overall reduction in chromatin accessibility at gene regulatory regions as a function of development. This is associated with a drop in the level of transcription factors (TFs) and in the transcription of genes needed for axonal regeneration [18, 19], and it might explain why forced expression of TFs or regeneration associated genes (RAGs) [20], alone or in combination, has resulted in limited axonal regeneration so far [21–23]. Although some experiments are biased by the heterogeneity of the nervous system tissue, there is evidence that expression of epigenetic modifiers and epigenetic marks are regulated during development. For example, immunoblotting of H3K9/14ac from cortical and cerebellar granule neuron (CGN) extracts [24] as well as H3K18ac and p300 immunostaining in retinal ganglia neurons (RGNs) [25] has revealed a decrease in the expression of these positive epigenetic marks in mature compared to earlier developmental stages. A chromatin immunoprecipitation small screening of histone post-translational modifications (HPTM) in cortical neurons revealed pronounced changes in histone marks at the promoter regions of several RAGs, indicative of progressive reduced chromatin accessibility during post-natal development [19].

Given the importance of chromatin structure for the accessibility of TFs to gene promoters and other gene regulatory elements, epigenetic mechanisms that control chromatin remodelling are strong candidates to play a crucial role in axonal regeneration. Here we provide a critical discussion of the role of epigenetic-dependent transcriptional mechanisms in mammalian axonal injury and we suggest future research directions.

## Epigenetic Mechanisms

Epigenetic modifications underpin changes in gene expression without alteration of the DNA sequence. In eukaryotes, DNA is organised in a hierarchical structure called chromatin, where its first order organisation is determined by the wrapping of DNA around histone proteins into nucleosomes, while the secondary and tertiary structures are stabilized and maintained by architectural proteins and chromatin remodelling factors [26, 27]. The degree of chromatin compaction plays a critical role in gene regulation, with highly transcribed genes and active regulatory elements, such as promoters and enhancers, being associated with a more relaxed and more accessible chromatin configuration [26–28]. The dynamic equilibrium between compact vs relaxed chromatin state in response to different stimuli is the result of multiple mechanisms, such as DNA methylation and hydroxymethylation, various HPTMs, exchange of histone variants, binding or displacement of architectural proteins, including ATP-dependent remodelling factors [29–31]. Nucleosomes are formed by the wrapping of 147 bp of DNA around a histone octamer, consisting of 2 units each of H2A, H2B, H3 and H4, stabilized by the association with H1 linker histone [32]. The presence of nucleosomes generally inhibits transcription, due to DNA bending or steric hindrance reducing the availability for transcription machinery to DNA binding [33, 34]. However, chromatin compaction and accessibility can be modified by the modifications of specific residues at the N-terminal tail of histones, affecting their interaction with the DNA. These modifications, including acetylation, methylation, phosphorylation, ubiquitination and SUMOylation, are the result of the balance between chromatin erasers and writers, which are enzymes responsible for removal or addition of such modifications, respectively, as reviewed elsewhere [35]. According to the so-called ‘histone code’ hypothesis, while histone modifications affect chromatin conformation, they can also indirectly exert their influence on gene transcription, via the recruitment of specific chromatin readers.

Acetylation at the ε-amino group of specific lysine (K) residues is the best characterized HPTM, and it is generally associated with transcriptional activation, providing binding sites for bromodomain proteins [36]. It is regulated by the balance between the activity of histone acetyl transferases (HATs) and histone deacetylases (HDACs) [35, 37]. HATs belong to three major families: general control non-derepressible 5 (Gcn5)-related N-acetyltransferases (GNATs), p300/CREB binding protein (CBP), and MYST [35]. The GNAT family includes the p300/CBP associated factor (PCAF). HDACs belong to four classes: class I-IV. All HDACs are zinc-dependent enzymes, except for class III HDACs, or sirtuins, which are NAD^+^-dependent enzymes, and constitute a structurally separate family [35]. Class I HDACs are ubiquitous proteins with high deacetylase activity toward histones, while class II HDACs display tissue expression specificity, prominent nucleo-cytoplasmic shuttling and a weak enzymatic activity, which *in vivo* is given by their association to class I HDACs [38].

Another level of epigenetic control is determined by DNA methylation. DNA methylation involves the covalent transfer of a methyl group to the C-5 position of the cytosine by DNA methyltransferases (DNMTs) [31, 39]. DNA methylation is regulated by a family of DNMTs: DNMT1, DNMT2, DNMT3A, DNMT3B, and DNMT3L [40]. While DNMT1 is generally involved in maintenance of methylation, copying DNA methylation patterns to the daughter strand during DNA replication [41], DNMT3A, DNMT3B, with the assistance of DNMT3L, are mainly responsible for *de novo* DNA methylation [42]. In mammals, more than 98% of DNA methylation occurs on CpG dinucleotides in somatic cells, while non-CpG methylation is more abundant in embryonic stem cells and brain tissue, where neurons show higher levels than glial cells [43, 44]. The CpG methylation pattern is found throughout the genome, with the exception of short unmethylated regions called CpG islands, mostly coinciding with promoters and first exons of protein coding genes [45–47]. Conventionally, methylation of DNA exerts a repressive role on gene transcription, acting at different levels. It represents a physical impediment to the binding of TFs, and more importantly, it is a docking point for methyl CpG binding domain (MBD) family proteins. These proteins are able to interact with a large variety of other factors, including HDACs, histone methyltransferases, polycomb complexes, and ATP-dependent chromatin remodelling factors, altering the histone code, influencing nucleosome stability and positioning as well as chromatin high order structure [31, 47, 48].

DNA demethylation has long been thought to be a passive event associated to the loss of 5-methylcytosine (5 mC) during successive rounds of replication, whereas active removal of the methyl group from 5mC has been controversial until the discovery of the ten eleven translocation (TET) enzymes, TET1, TET2, and TET3 [49–51]. TET enzymes catalyse the consecutive oxidation of 5 mC 5-methylcytosine (5mC) to 5-hydroxymethylcytosine (5hmC), 5-formylcytosine (5fC), and 5-carboxylcytosine (5caC) [52]. The thymine DNA glycosylase (TDG) can subsequently excise TET oxidation products, leading to the complete DNA demethylation [51]. While 5mC is found in all mammalian tissues, corresponding to almost 4-5% of all cytosines, 5hmC level is lower and displays a tissue specific pattern. It is prevalent in embryonic stem cells and in the brain where it can reach about 40% of the total 5mC, while it is rare in the spleen and testes (only about 0.03%–0.06%) [49]. Nevertheless, this modification, along with 5fc and 5ca, is thought not to be a mere transient intermediate of the DNA demethylation enzymatic process but to be persistent in time and to facilitate gene expression [53]. Interestingly, in the mouse and human brain, 5hmC is enriched at genes involved in synaptic function [54].

## Histone Acetylation: HDAC Inhibitors, HATs and HDACs in Axonal Injury and Regeneration

### HDAC Inhibitors

The importance of histone acetylation in axonal regeneration has initially been suggested by experiments with broad range HDAC inhibitors (HDACi), such as pan-HDACi trichostatin A (TSA) in cultured rat CGN. TSA increased acetylation of H3 at K9/14, neurite extension and growth cone remodelling and this required transcription, since transcriptional inhibitors blocked TSA-dependent neurite outgrowth [24]. The effect was observed on both growth permissive and non-permissive substrates such as myelin, in line with other evidence showing that treatment with class I and II HDACi Valproic acid (VPA) allowed cultured embryonic spinal cord and hippocampus neurons to partially overcome growth inhibition by the myelin associated protein NogoA. Interestingly, VPA enhanced recovery of locomotion in rat spinal cord contusion model, however the mechanisms underlying this recovery had not been explored [55, 56]. Subsequently, it has been reported that TSA injection into the vitreous at the time of an optic nerve crush (ONC) in rats increased RGN survival and H3K18ac levels, although it failed to enhance axonal regeneration [25]. Systemic injection of TSA or of class I HDAC inhibitor MS-275 led to a global increase of acH4 in dorsal root ganglia (DRG) neurons and specifically at the promoters of several RAGs. This also resulted in increased RAG expression and in enhanced neurite outgrowth of *ex vivo* cultured DRG neurons [57]. Since the positive effect on neurite outgrowth was observed up to 2 weeks after the last injection of MS-275, when the level of histone acetylation at the RAG’s promoters had come back to baseline, this suggests a long-lasting increase in acetylation on other target genes or non-histone acetylation dependent mechanisms. However, the *in vivo* treatment with MS-275 limited axonal die-back but did not result in significant axonal regeneration of DRG sensory fibers beyond the spinal injury site [57]. Despite the lack of selectivity towards individual HDACs and/or to the lack of cellular specificity, these findings suggested that the inhibition of HDACs leading to an increase in histone acetylation might be beneficial for axonal regeneration. Indeed, using DRG pseudo-unipolar neurons as a model to compare molecular responses between a regenerative peripheral vs non-regenerative central axonal injury [58] within the same cell body, it has been found that acH4 level increased 6h after a peripheral, but not a central axonal injury. This occurred both globally in DRG neurons and on the promoters of a few tested RAGs [57]. Accordingly, in a low throughput screening of HPTMs 24 hours following axonal injury, Puttagunta et al. [59] showed that H3K9ac level increased upon sciatic nerve axotomy (SNA) in DRG neurons together with H3K9ac occupancy on the promoter of selected RAGs, such as Galanin, Gap43, and Bdnf. On the contrary, H3K9ac levels decreased upon dorsal column axotomy (DCA), while the repressive mark H3K9me2 was enriched on the same promoters. These changes correlated with gene expression, although not for all of the tested RAGs. These studies suggested the presence of a differential chromatin state upon these two types of injury and that the increase of each histone mark is only partially correlated to the activation of the entire transcriptional programme, pointing to the need for more systematic high throughput studies.

### HATs

Several studies have identified CBP/p300 and PCAF as HATs that play important roles in neurite outgrowth and axonal regeneration. Using small interfering RNA (siRNA) approaches Gaub et al. [24] identified CBP/p300 and PCAF as responsible for TSA-induced H3K9/14ac and neurite outgrowth in CGN. Neurite growth in control cells was not significantly affected on permissive substrates, suggesting a compensation between these enzymes, or that a minimal level of expression might suffice for neurite outgrowth in permissive growth conditions. Q-PCR and ChIP-qPCR experiments in TSA treated CGN indicated that TSA induces increased CBP/p300 and PCAF expression correlating with higher H3 acetylation level on their promoters. Although those experiments had the bias of glial cell contamination, the result was supported by the correlation between single cell CBP/p300 expression, H3 acetylation level, and neurite outgrowth in TSA treated cells. A similar analysis in control vs treated cells, and qPCR from a pure neuronal population would have helped clarify whether TSA is actually responsible for the increased expression of CBP/p300 and PCAF via increased histone acetylation at their promoters. However, overexpression of CBP/p300 and PCAF in CGN by electroporation was sufficient to enhance neurite outgrowth in permissive and inhibitory substrate to an extent comparable to TSA treatment.

In follow up studies Gaub et al. [25] showed that the failure of TSA in promoting axonal regeneration following ONC correlated with the lack of induction of p300 expression. Accordingly, adeno-associated virus (AAV) mediated p300 overexpression in the eye, via intravitreal injection, was sufficient to increase axonal regeneration in RGN after ONC, synergising also with lens injury, a conditioning lesion that enhances optic nerve axonal regeneration via inflammatory stimulation. Overexpression of p300 increased H3K18ac, the occupancy of p300 and acH3 on the promoter of growth-associated genes such as Gap43, Sprr1a, Coronin 1b that also displayed increased gene expression. P300 overexpression in RGN likely underpins cell autonomous effects upon axonal regeneration, although expression in amacrine and bipolar neurons was observed too.

In agreement with these findings [24], *in vitro* AAV mediated PCAF overexpression resulted in increased neurite growth on permissive and inhibitory environment on both cultured DRG and CGN. This led to increased expression of selected RAGs, while the PCAF inhibitor Garcinol inhibited neurite outgrowth and regenerative gene expression [59]. Remarkably, a single Garcinol intrathecal injection just before SNA, reversed the conditioning-dependent increase in DRG neurite outgrowth. Since Garcinol can also inhibit p300/CBP, which are involved in axonal regeneration [25], the authors confirmed the specificity of these results by using PCAF null mice. DRG neurons from PCAF null mice failed to display regenerative growth both in culture and *in vivo* after a conditioning lesion and spinal cord injury, although the use of a neuronal specific PCAF knock out (KO) would have been advisable to distinguish between neuronal vs non-neuronal specific effects. More importantly, *in vivo* PCAF overexpression in DRG neurons mediated by AAV was sufficient alone to induce increased level of H3K9ac and increased expression of RAGs. PCAF overexpression promoted sensory axonal regeneration up to 1 mm from the lesion site in a T9-T10 spinal dorsal column crush, mimicking and surpassing the regenerative ability of a conditioning lesion. Although it is difficult to compare different studies because of different spinal cord injury (SCI) models, and despite functional tests have not been performed, it is worth noting that the degree of axonal regeneration achieved in this study was higher than the one obtained with MS-275 injection after a C5 dorsal column axotomy [57]. This possibly reflects the fact that MS-275 was able to mimic the regenerative transcriptional programme only partially but it also suggests that epigenetic changes on key genes, working as functional hubs in neurons [60], may be better able to elicit axonal regeneration compared to global, less specific, changes driven by HDACi. In this regard, high throughput studies will be necessary to fully characterize the role of PCAF on gene promoters and enhancers.

An interesting biological question is how HATs are modulated after axonal injury. Puttagunta et al. demonstrated for the first time a link between retrogradely transported signalling molecules and epigenetic modifications [59]. They showed that activation of PCAF and its binding to RAG promoters was ERK dependent, whose phosphorylation and retrograde transport increased following injury [61, 62]. Indeed, nerve treatment with a MEK1/2 inhibitor abolished the SNA dependent increase in the phosphorylation of PCAF, H3K9ac level globally in DRG nuclei and specifically on the RAG promoters. It also abolished *ex vivo* neurite outgrowth, whereas NGF treatment, activating pERK signalling, mimicked the injury dependent increase in PCAF phosphorylation.

### HDACs

The very first molecular events following a peripheral axonal injury are represented by changes in electrical activity and Ca^++^ influx [63, 64] that lead to an increase in cAMP levels [65, 66] and activation of multiple signalling cascades [2, 62, 67, 68]. Cho et al. [69] showed that a peripheral axotomy induced the nuclear export of HDAC5 towards the periphery in DRG. This relied upon Ca^++^, protein kinase C (PKC) and microtubule cytoskeleton. HDAC5 export increased tubulin deacetylation and acH3 level in the nuclei of DRG neurons, contributing to growth cone dynamics and axonal regeneration [70]. Taking advantage of a mutant form of HDAC5 that cannot be transported but it is entrapped in the nucleus (HDAC5nuc), they demonstrated that the HDAC5 nuclear export is needed for axonal regeneration in culture and for the expression of a regenerative transcriptional programme. Indeed a gene microarray study at 3-8-12 h upon *in vitro* axotomy in GFP and HDAC5nuc expressing DRG neurons identified injury responsive genes dependent from HDAC5 nuclear export. While injury responsive genes were more abundant at 8 h upon axotomy, the ones dependent from HDAC5 were more represented at 3 and 12 h, reaching about 50% of the transcriptional programme, pointing to a role for HDCA5 in controlling early response genes. HDAC5-dependent gene expression included functional categories related to transcription, cell adhesion, and signalling. In line with this, subcutaneous injection of I3A, an activator of PKC, was able to mimic and synergize upon DRG regenerative growth with the sciatic nerve conditioning lesion in *ex vivo* cultured DRG. I3A delivery was also able to enhance HDAC5-dependent axonal regeneration *in vivo* upon nerve crush. Since class II HDAC enzymatic activity is low compared to other HDACs, and HDAC4 and HDAC5 have been shown to interact with HDAC3 [38, 71], the authors confirmed that HDAC5 controls the subcellular distribution of HDAC3 and its nuclear export upon axonal injury in DRG neurons. Therefore, further studies will be necessary to distinguish between HDAC5 direct vs indirect, possibly via HDAC3, effects on gene expression. This work proposes HDAC5 and HDAC3 as interesting candidates to be tested in models of CNS injury, where HDAC5 and HDAC3 nuclear export may fail to happen, as suggested by studies in RGN [70, 72]. Recently, HDAC3 inhibition has been shown to have beneficial effect on functional recovery and myelination upon SCI and nerve injury, acting on the polarization of microglia/macrophages and Schwann cells respectively [73, 74]. This does not exclude additional roles in neurons, since a neuroprotective role of HDAC3 inhibition has been proven in RGN [75, 76]. Similarly, combined genetic ablation of Hdac1 and 2 has recently been shown to promote neuroprotection following optic nerve axotomy via p53-dependent pathways [77]. HDAC2 genetic or pharmacological inhibition has also been found to promote recovery of motor function, to enhance cell survival and neuroplasticity as well as to reduce neuroinflammation in a stroke model in rodents [78, 79].

## Non-histone Acetylation in Axonal Injury and Regeneration

HATs and HDACs can have additional targets other than histone proteins [80], and some of those non-histone acetylation events have been linked to axonal regeneration. For example, p300/CBP and PCAF can mediate axonal regeneration also via acetylation of p53 on K373/320 and C/EBP [24, 25, 81]. However, further studies are needed to discriminate between histone acetylation vs non-histone acetylation contribution of p300 or TSA mediated axonal regeneration. The data seems to imply that in these paradigms the contribution of the acetylation of p53, C/EBP or of other TFs might overcome histone acetylation-dependent axonal regeneration since (i) the overexpression of a mutant form of p53 that cannot be acetylated almost completely abolished neurite outgrowth in control and TSA treated CGN; (ii) TSA treatment, which did not affect acetylation of p53, failed to induce axonal regeneration. Lastly, active acetylated p53, following MDM2/4 inhibition, enhanced axonal sprouting and regeneration following spinal cord injury irrespective of histone acetylation [82].

Finelli et al. [57] described a positive feedback between histone acetylation and the function of SMAD1, a TF previously identified to be involved in axonal elongation [83]. They found an inverse correlation between pSMAD1 occupancy and HDAC1 binding at four gene promoters (Sprr1a, Galanin, Vip, Npy) and a positive correlation with p300 binding at Sprr1a promoter in Neuro2A cell lines. Moreover, pSMAD1 was found to interact with p300 in a lysate of cultured DRG. These studies have the limitations to have been performed in Neuro2A cell lines or in cultured DRGs, therefore whether such a complex on DRG derived chromatin exists *in vivo* remains unclear and it deserves further investigations. Nevertheless, it suggests that SMAD1, similarly to SMAD2 and SMAD3 [84], can be a target of p300-dependent acetylation.

Injury dependent cytoplasmic export of HDAC5 led to decreased level of acetylated tubulin, which correlated with microtubule stability. In this way, HDAC5 controls microtubule dynamics in injured DRG neurons, improving growth cone remodelling and regeneration [69]. Other studies indicated that HDAC6 inhibition too can mediate both neuroprotection and axon growth on myelin substrate via α-tubulin acetylation [85]. This apparent contradictory result can be explained by the different experimental conditions including an axotomy in the HDAC5 study and growth on inhibitory substrates for HDAC6. Additionally, HDAC6 is known to deacetylate, among other proteins, cortactin, which is required for actin and growth cone remodelling [85–87].

Together, non-histone acetylation deserves further investigation, since acetylation/deacetylation of several TFs or signalling molecules, such as STAT3, c-myc, SMAD7, NF-Kb, HIF1a, FOXO1, E2F, phosphatase and tensin homolog (PTEN), many of which are involved in axonal regeneration, has been shown to affect their stability and/or their activity [80, 88, 89], thus potentially impacting the axonal regenerative ability.

## DNA Methylation and Hydroxymethylation in Axonal Injury and Regeneration

Using a combined spinal cord and conditioning sciatic nerve injury in rats, it was found that axonal injury induces increased expression of the folate surface receptor 1 (FLR1), as shown *by in situ* hybridization and immunohistochemistry in DRG cell bodies and by qPCR in the spinal cord [90]. FLR1 was required for DRG neurite outgrowth *in culture*, since DRG neurite outgrowth was significantly impaired in Flr1^+/-^mice. Moreover, folate supplementation was able to promote axonal regeneration in CNS injury models, such as optic nerve and dorsal column cervical transection, however this did not occur in Flr1^+/-^ mice. Since axonal injury was accompanied by decreased DNMT3a and DNMT3b protein levels and decreased DNA methylation in the spinal cord, folate supplementation seems to counteract the injury-dependent hypomethylation, by restoring the level of DNMT3a and DNMT3b. Indeed, folate supplementation affected global methylation levels and specific methylation of the Gadd45a promoter in the spinal cord, and the DNMT antagonist 5-azacytidine (5-AzaC) partially blocked folate-dependent spinal axonal regeneration *in vivo* [90]. However, since most of the assessments were carried out in bulk spinal cord tissue, it remains to be clarified whether this reflects an effect on neuronal or glial cells. Use of neuronal specific Flr1 knock down (KD) mouse models and/or cell specific assessment of the DNA methylation and DNMT levels would have been helpful in this regard. The consequence of folate intake on the levels of DNA methylation, and expression of DNMTs and MDMB proteins is well established. Although the mechanisms are still unclear, it is one of the interesting examples of how nutrition/diet may affect the epigenome [91, 92]. A later study [93] showed the cell specific expression of DNMTs in rat DRG neurons by immunohistochemical analysis. While DNMT1 was expressed in both glia and neurons, DNMT3a was preferentially expressed in glia and DNMT3b was preferentially expressed in neurons, although the use of specific glial and neuronal subtype markers would have complemented the analysis to identify possible different expression patterns among glial and neuronal cell types. Using a nerve ligation model of neuropathic pain, qPCR studies revealed a robust increase of DNMT3a expression only at one week upon injury, which was sustained up to four weeks. However, DNMT1 and DNMT3b increase was weaker and delayed weeks after ligation. These studies seem at odds with earlier investigations revealing that DNMT1 and DNMT3a were highly expressed in adult post-mitotic neurons, whereas DNMT3b was poorly expressed [94–96]. However, together with Iskandar et al. [90], they indicate that DNA methylation could be affected following axonal injury.

The first attempt to answer this question with a high throughput approach, checking for promoter and CpG island specific changes, was a microarray study performed comparing DNA methylation pattern in mouse DRG at 1, 3, and 7 days upon a peripheral sciatic nerve axotomy or a central dorsal column spinal axotomy [59, 97]. Although only a modest number of genes were found to be differentially methylated with respect to sham injury and none was associated to RAGs, the methylation temporal dynamics was distinct in the two conditions. Hypermethylated genes were prevalent at 1 day after SNA, while hypomethylated genes peaked at 7 days upon DCA. More interestingly, differentially methylated genes related to transcription and chromatin remodelling were well-represented upon SNA but they were hardly found among hypomethylated genes following DCA. Following nerve axotomy, ion channels were clearly more abundant among hypermethylated genes with respect to hypomethylated, and this is interesting since ion channels have been found to be regulated following injury and linked to axonal extension and regeneration [98–101]. Nevertheless, this study has the limitation to be biased for promoters and CpG islands of the 18,000 genes represented in the arrays, and the whole DRG used contained both neurons and glial cells. Further genome wide studies are necessary to characterize the DRG DNA methylome upon axonal injury, possibly at the single cell level.

Recently, 5hmC has deserved much attention in cell biology. Both Loh et al. and Weng et al. [102, 103] found that peripheral nerve injury results in increased level of TET3, but not TET1 and TET2, and 5hmC in DRG. TET3 increase was counteracted by the use of Ca^++^ chelators [102], suggesting that injury-dependent increase in calcium signaling might be required for 5hmC. Moreover, TET3 KD by AAV mediated short hairpin (shRNA) abolished nerve injury dependent increase of 5hmC [102]. Using a next generation sequencing (NGS) approach in DRGs following 5hmC capture, Loh et al. showed changes in 5hmC pattern at unique genomic locations upon peripheral and central axotomy with respect to naïve tissue [103]. Surprisingly, a higher number of differentially hydroxymethylated regions (DhMRs) upon DCA, with a bias for a gain in 5hmC, with respect to SNA was described. About 40% of the RAGs previously identified by the same group by microarrays [83] presented differential hydroxymethylation, but a clear correlation with their expression was not evident. This poor correlation might be affected by the limitation of comparing microarrays vs next-generation sequencing studies. Additionally, approximately half of DhMR-associated RAGs contain multiple DhMRs, often displaying opposite patterns and the majority of the DhMR occurred at ‘open sea’ regions. Interestingly, signalling pathways and TF motif analysis of the peripheral and central DhMRs revealed association to different repertoires of signalling molecules and TFs. For example, IRF3/5 and STAT families were specifically associated to peripheral DhMRs, ARI3A, FOX, and MAZ were specifically associated to the central DhMRs, whereas HIF1A and ARNT were present in both. Some of those TFs have been associated to transcriptional response to axonal injury and/or axonal regeneration [104–106], whereas others, such as FOX and ARI3A, are interesting candidates for further studies, since they have been linked to chromatin remodelling [107–109].

In line with these findings, Weng et al. [102] found that TET3 KD by AAV mediated shRNA reduced the number and length of DRG neurites *in culture* and nerve regeneration *in vivo*. This was not due to cell loss as demonstrated by caspase immunostaining. While WT animals showed signs of skin re-innervation and progressive functional recovery at the heat-evoked hind paw withdrawal test, TET3 KD mice showed significantly impaired responses. Q-PCR and immunohistochemistry analysis revealed that TET3 KD attenuated the nerve injury induced expression of several RAGs, such as ATF3, STAT3, SMAD1, and c-myc, with SMAD1 and STAT3 expression being impaired also in naïve condition. Performing targeted bisulfite sequencing from a neuronal purified preparation the authors proved that nerve injury induced TET3 occupancy and DNA demethylation at enhancers and gene bodies of specific RAGs, and that this was lost in TET3 shRNA transduced neurons. Interestingly, in a model of regenerating CNS axons following PTEN deletion in RGNs [110], TET1 was required for nerve regeneration. Further studies are needed to address the regulation of TET proteins upon axonal injury. Little is known about post-translational modifications of TET enzymes. They can be phosphorylated by JNK, PKC, and ATM resulting in changes in their cellular localization, protein stability and function. Phosphorylation can be suppressed via *O*-GlcNAcylation by the glycosyltransferase OGT [111, 112]. Interestingly, TET proteins, by recruiting OGT to chromatin, can also participate in *O*-GlcNAc modification of histones, a newly discovered HPTM linking nutrient availability to chromatin remodelling [113]. Another outstanding question that needs to be addressed is the contribution of the loss of DNA methylation vs the increase of DNA 5hmC in axonal regeneration. Weng et al. [102] found that the KD or KO of TDG, the enzyme responsible for the removal of TET oxidation products and complete DNA demethylation [51], impaired nerve regeneration and RAG expression in a similar way compared to TET3 KD. Intriguingly, TDG KD led to enhanced levels of 5hmC, suggesting that increased 5hmC level may not be sufficient for RAG expression and subsequent axonal regeneration, but rather that complete DNA demethylation must occur. This might also partially explain the lack of consistent correlation between 5hmC changes and RAG expression in the Loh study [103]. It would be therefore interestingly to correlate 5hmC changes with DNA methylation levels during a time course upon axonal injury. This would allow evaluating whether a higher association with gene expression can be found for genes showing DNA demethylation following an increase in 5hmC.

## RNA Mediated Regulation in Axonal Injury and Regeneration

Several studies have uncovered the role of non-protein coding RNA transcripts, such as microRNAs (miRNAs), small interfering RNAs such as microRNAs (miRNAs), siRNAs, Piwi-interacting RNAs (piRNAs), and long non-coding RNAs (lncRNAs), in the control of gene regulation via epigenetic mechanisms. LncRNAs mediate gene silencing by recruiting Polycomb proteins, histone methyltransferases, and DNMTs, and they may display enhancer like function [114]. Many miRNAs can control gene expression by affecting expression of epigenetic remodellers, such as Polycomb proteins, HDACs, DNMT3a, p300. There is evidence that miRNAs and lncRNAs are differentially expressed in the neural tissue after various types of injury, such as nerve, spinal cord, traumatic and ischemic brain injury, where they promote regeneration by regulating key biological processes, including cell survival/apoptosis, extracellular matrix-cytoskeleton signalling, and neurite outgrowth [115]. Therefore, an interesting line of research would be investigating whether epigenetic changes via ncRNA also regulate axonal regeneration. A few studies seem to indicate that it might be a possibility. For example, a mutual feedback loop between miR-138 and SIRT1 has been described, which contributes to axonal regeneration. Sirt1 is the target of miR-138 in DRG neurons, and in uninjured neurons the high level of miR-138 suppresses the expression of SIRT1, contributing to the low intrinsic axonal regeneration ability. Upon nerve injury, down-regulation of miR-138 correlates with an increase in Sirt1 levels, which is required for axonal regeneration and it also acts as a transcriptional repressor to directly suppress the expression of miR-138 [116]. As another example, miR-206 is upregulated in a mouse model of ALS, and upon nerve injury in wild type mice, where it is beneficial for axonal regeneration. It regulates HDAC4 via transcriptional repression, which might promote axonal regeneration as suggested by mice lacking HDAC4 that showed faster muscle reinnervation after axonal injury [117, 118].

mRNA post translational modifications, constituting the so called epitranscriptome, contribute to the regulation of gene expression as well. Among them, N-6-methyladenosine (m^6^A) is the most abundant and has been implicated in mRNA stability and processing, export, and translation [119, 120]. Intriguingly, a recent report [121] has demonstrated that peripheral nerve injury is associated with an increased level of m6A in DRG neurons, with a peak at 1-3 days upon injury. Using m^6^A-SMART-seq the authors found that more than 6000 transcripts were m^6^A tagged, and about 182 were upregulated at 24 h upon sciatic nerve injury. Among them, about 30 RAGs were identified, such as Atf3, Gadd45, Sox11, and Tet3. Interestingly, gene ontology analysis revealed enrichment of ribosomal and translation machinery proteins. Using m^6^A-CLIP-SMART-seq, having single base resolution, the m^6^A sites were found enriched in start sites, stop codons, and exons. Interestingly, RAG transcripts displayed the larger gain in m6A, and in some of them new sites were found, mostly in exons. Neuronal deletion of Methyltransferase like 14 (Mttl14), the enzyme responsible for such modification, did not result in significant effect on gene expression in naïve or injury conditions, whereas it strongly impaired injury dependent *de novo* protein synthesis, in accordance with the proposed role of RNA m^6^A [120]. This led to impaired nerve regeneration and functional recovery at the heat-evoked hind paw withdrawal test *in vivo*, without affecting neuronal death. A similar effect was observed upon the KO of the Ythd1 factor, a protein involved in the facilitation of translation of m^6^A tagged mRNA [122]. More importantly, Mettl14 KD resulted also in impaired RGN survival and axonal elongation in a model of PTEN deletion-induced axonal regeneration upon ONC. Since Mttl14 KO did not affect global gene expression levels, and m^6^A tagged transcripts were enriched for translation machinery proteins, this works raises the intriguing hypothesis that m^6^A tagging promotes global protein synthesis upon injury required for sustained axonal regeneration. Therefore, the investigation of m^6^A induced protein repertoires upon peripheral and central axonal injury represents a new promising field of investigation.

**Fig. 1:**
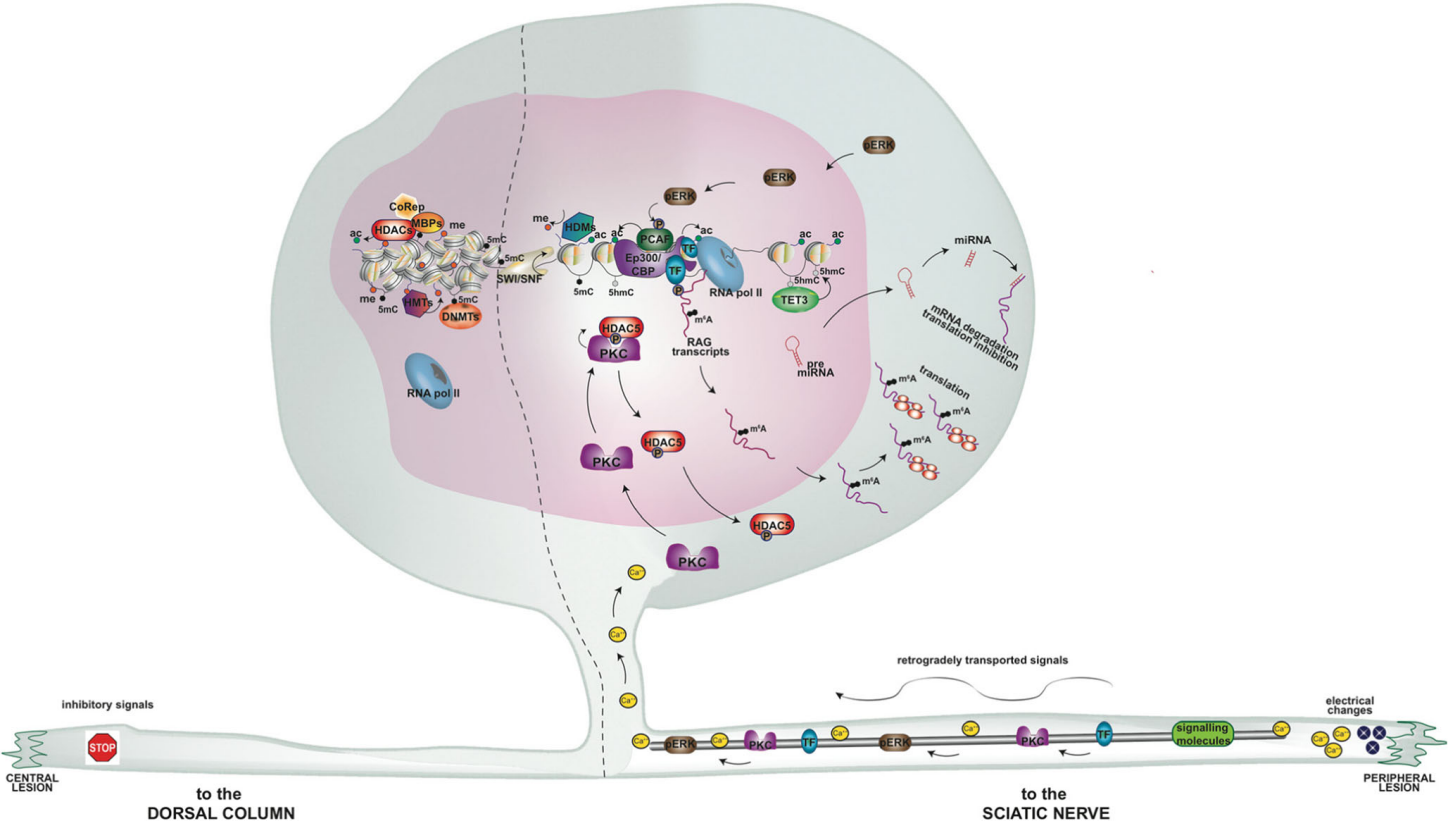
Cartoon summarizing epigenetic pathways upon an axonal injury in the peripheral and central nervous system, based upon *in vivo* and *in culture* experimental evidence. A dorsal root ganglia neuron is schematized with a peripheral branch towards the sciatic nerve and a central branch towards the dorsal column. Upon a nerve injury (on the right) changes in electrical activity and Ca^++^ influx result in activation of signalling molecules (ERK and PKC among them) and transcription factors (TFs), which are retrogradely transported to the soma. PKC leads to phosphorylation and nuclear export of histone deacetylase 5 (HDAC5), while pERK leads to phosphorylation and activation of p300/CBP associated factor (PCAF), resulting in increased histone and TF acetylation (ac) at the gene promoters of regeneration associated genes (RAGs). Calcium signalling is also responsible for the DNA hydroxymethylation (5hmC) mediated by Ten Eleven Translocation 3 (TET3) at the RAG gene bodies and enhancers. Chromatin remodelling factors, eg. SWItch/Sucrose NonFermentable (SWI/SNF), might also play a role. Altogether, this results in increased RAG transcription, via RNA polymerase II (RNA pol II). Some transcripts can be modified by m^6^A addition, leading to increased translation efficiency. On the contrary, HDAC transcripts can be miRNA targets, representing positive feedbacks for sustained regeneration. Upon a dorsal column injury (on the left part) activation of inhibitory signalling pathways does not allow epigenetic remodelling. DNA methylation (5mC) and histone methylation (me) recruit silencing factors, eg. Methyl binding proteins (MDMs), histone deacetylase (HDACs), histone methyltransferase (HMTs), and nuclear corepressor (CoRep)

The key signalling pathways relying on epigenetic control of axonal regeneration described in this manuscript have been summarised in a diagramme (Figure 1).

## Limitations and Further Perspectives

So far, only limited regeneration has been achieved by TF overexpression, pharmacological or genetic modulation of epigenetic modifiers. The heterogeneity of the nervous system is a challenge to the investigation of neuronal vs non neuronal mechanisms and especially so in case of very rare biological events, such as in the case of DNA 5hmC. Moreover, very few systematic unbiased studies have directly compared and correlated transcriptional changes and epigenetic modifications specifically in a peripheral vs central axonal injury paradigm, in the attempt to identify which epigenetic modification/s might be able to reprogram CNS neuron in a regenerative state [59, 97, 103, 106]. So far only DNA methylation and hydroxymethylation profiles have been systematically assessed, although DNA methylation was limited by the depth of the microarrays employed [59, 97, 103].

Moreover, at the moment, only histone acetylation and partially methylation have been investigated. However the histone code is much richer, including phosphorylation, ubiquitinylation, SUMOylation, ADP ribosylation, and deamination [35]. Moreover, modifications of the histone globular domains have been described, associated to nucleosome assembly and transcription regulation [123]. Interestingly, lysine acylation, including propionylation, butyrylation, succinylation, malonylation, crotonylation, and glutarylation has been recently described to be associated to either globular or tail domain [123]. Acyl-coA groups used for these modifications derive from metabolic reactions, such as fatty acid β oxidation, citrate cycle, and the aminoacid metabolism, suggesting a metabolic control of the epigenome. Since metabolic responses upon brain injury have been described [124], it would be interesting to assess whether metabolic changes might affect axonal regeneration via epigenetic remodelling. Next, in addition to HPTM, histone turnover of the highly dynamic H3.3 histone variant has been associated to neuronal transcription and plasticity [125], and the role in axonal regeneration remains unknown.

Chromatin high-order structure, determined by the action of chromatin remodellers and architectural proteins, is an important feature in gene expression regulation [27, 126]. Chromatin remodellers, belonging to the SWItch/Sucrose NonFermentable (SWI/SNF), Imitation SWI (ISWI), Nucleosome Remodelling and Deacetylase (NURD/Mi-2/CHD), and Inositol Auxotroph 80 (INO80) families, are motor proteins that use the energy of ATP hydrolysis to alter nucleosome structure, affecting transcription initiation and elongation, histone variant deposition, DNA repair [29–31]. Architectural proteins, such as CCCTC-binding factor (CTCF) and cohesin, play a major role in genome organization by their ability to assist in the formation of long-range contacts between genomic loci via chromatin loops, including between promoters and enhancers, facilitating transcription [127–129]. Although changes in chromatin structure have been involved in several aspects of neuronal biology and pathology, such as neurogenesis, neuronal differentiation and maturation, dendrite morphogenesis and growth, cognition, neurodevelopmental and neurodegenerative disorders [127, 130–136], it is not known if they might play a role in axonal regeneration.

Genome wide extensive studies, such as Chromatin immunoprecipitation (ChIP)-, Assay for transposase accessible chromatin (ATAC)-, micrococcal nuclease (MNase)-, bisulfite (BS), Hi capture sequencing are necessary to complete the picture of the epigenetic modifications, the chromatin conformation and structural changes occurring upon axonal injury, and to understand how all these cooperate in driving or hindering axonal regeneration. The epigenetic studies discussed so far have the limitations of reflecting a mixed neuronal, glial and at times immune cell populations of cells. Performing these assessments in specifically enriched cell populations will be fundamental to dissect the contribution of neurons vs glial or infiltrating immune cells to axonal regeneration. A further refinement would be the use of single cell epigenome sequencing technologies [137] that will address possible neuronal type specific differences in axonal growth potential.

Ultimately, these studies could pave the way to the ambitious goal to reprogram neurons into a regenerative state, in a timely controlled and specific manner, taking advantage for example of novel tools such as CRISPR/Cas9 genome editing.

## Electronic supplementary material

ESM 1 (PDF 510 kb)
